# A Case of Petrifaction

**Published:** 1869-05

**Authors:** 


A Case of Petrifaction.—The following singular case of petrifaction was recently published in the *Criminal Zeitung* of Dec. 4th, 1868.

Amos Broughton, of Wayne County, Iowa, died six years ago, and recently on disinterring the body it was found in a state of petrifaction, like a marble statue. Every feature was perfect, and the whole face life-like. The weight of the statue was 400 pounds. Broughton weighed just before death 200 pounds.

